# Alcoholic

**Published:** 1877-07

**Authors:** 


### ALCOHOLIC.

Temperance has received a new impetus with the advent of Francis Murphy. One fails to appreciate the wide prevalence of alcoholic intoxication until some popular movement, like the present one, enlists our interest and attention.

We can bear witness that much good has been wrought in our city through the efforts of a few earnest and enthusiastic men, yet very much remains to be accomplished ere all our inebriates are reclaimed, for we notice the young men frequenting the saloons almost as numerously as before, while our streets scarcely show any diminution of reeling tipplers.

The moral aspect of intemperance is abundantly preached, while the medical bearing of the vice is seldom broached. We desire to fire off our little squib into the mass of verbosity that the occasion has inspired. It would seem that, could the effects of alcohol upon the system be fully understood by our young men, they would be unwilling to submit themselves to its poisonous and seductive influences. We are sure that the dileterious consequences of introducing alcohol into the stomach are not appreciated, else would not the danger be continually invited.

The beverages usually partaken of are brandy and whisky, containing from 48 to 56 per centum of alcohol. Port wine contains, in every hundred ounces, 22.96 ounces; Madeira, 22.27; Sherry, 19.17; Claret, 13.10; Champaigne, 12.61; Porter, 4.20; Ale, 8.88; Lager, 2. From this it is seen that in all these beverages, more or less alcohol is partaken of; lager beer, even, considered by many so harmless, contains quite a quantity of this harmful ingredient.

Now, when alcohol is taken into the stomach, its first effect is to coagulate all the albumen contained in the food, rendering it almost as indigestable as so much leather. If it is present in large quantity, it *cures* the meats and vegetable substances partaken of as food, in such a manner as to completely defeat the digestive function. Most persons are familiar with the fact that, the anatomical specimens usually met with in surgeon's cabinets are always preserved in alcohol. Flesh, can never change if kept in this fluid. When, therefore, it is partaken of at, or immediately after a meal, the stomach is converted into a sort of museum for the preservation of undigested substances. Not only this occurs, but the liquor excites the glands of the stomach to throw out an excess of the digestive juices, causing congestion, when the enlarged vessels absorb the alcohol, conveying it into the blood, from which source it is deposited largely in the brain, liver, kidneys and other important organs of the body. It is a matter of fact, demonstrated again and again by experiment as well as by casual observation, that alcohol is absorbed directly into the circulation of the blood, where it is at once capable of acting as a direct poison and irritant to the nervous centres.

Dr. Clymer, an eminent physiologist, instances a post-mortem of a man who, for years, was addicted to the drinking of brandy, in which every important organ of the body had suffered degeneration. The fluids collected from the cavities of the brain, contained 2.6 per centum, by volume, and 2.1 per centum by weight, of free alcohol.

Imagine the condition of a man's brain soaking in such a fluid as that! Why, you may take the brain of a calf, immerse it in alcohol for one week, when, instead of the soft, delicate structure with which we are all familiar, we will find a hard, dense, leathery substance that could be used for a foot-ball without danger of injury to any of its parts. Every physician is familiar with the fact that, in order to be able to handle a brain for dissection or examination, it first becomes necessary to place it in alcohol over night to harden it. Precisely the same process obtains in the habitual drinker, notably so in the moderate drinker, when, if he does not heed the warning that presents itself in the form of a deranged stomach and morning head-aches, soon drops into hopeless imbecility or paralysis from hardening of the brain. It is well known to the profession that the paralysis of middle-age—much of it—is directly attributable to habitual drinking, indeed, one-quarter of all male insane people, have paralysis, in some form, attributable to alcoholism or other intemperate habits. The effects of alcohol upon the system is cumulative, it being stored and added to from time to time as the drinker indulges in his depraved appetite. From this circumstance, a noted English physician has shown that a vast amount of morbid changes occur throughout the different organs of the body. From a large number of post-mortems which he has tabulated, we learn that disease of the brain caused the death of 92 per centum of the inebriates examined. Disease of the lungs existed in 63.24. Disease of the liver—fatty degeneration, waxy liver and the like—occurred next in frequency. Disease of the heart and large arteries, caused the death of many, while stomach troubles, congestion, ulceration, dyspepsia, &c., were numerous.

Experiments upon animals have demonstrated the fact that alcohol in the blood retards the circulation and stints the secretion of bile. This condition of affairs tends to produce fat—fat being detected floating in the blood, endangering the victim to sudden death. By coagulating the blood, it also subjects the person to irregularity of the heart and arrest of its function, and by producing congestion of the brain, it causes sudden death from apoplexy.

These dangers are all recognized by life insurance companies, who are very particular in their questions about intemperance, refusing the applications of all who are addicted to strong drink. Your physician recognizes the danger also, and when called to your bedside, inquires anxiously about your drinking habits, and from your inebriety arguing that the chances are against your recovery.

But, aside from the influences exerted upon health, by the use of alcohol, exists the far more important consideration of its effects upon our race! Chronic alcoholism entails upon the subject an enfeebled and diseased constitution. Causes disease of the nervous system, which does not expend itself alas, upon the hapless inebriate, but reaches out and beyond, and fastens itself upon the vitals of his progeny, causing epilepsy, idiocy and insanity!

Sir James Clark has clearly shown that drunkenness manifests itself in the fourth generation. In his interesting monograph upon the subject he relates the effect upon the 1st generation as being, alcoholic excesses, immorality and depravity; in the 2d generation, hereditary drunkenness, attacks of mania and general paralysis; in the 3rd, sobriety generally prevails, but hypochondriacis and homicidal tendencies were expressed; in the 4th, intelligence becomes feeble, mania is developed at 16 years of age, while stupidity and idiocy increases until the family becomes extinct.

What a fearful responsibility we take upon ourselves when we render our children liable to such an inheritance! Who among us are willing to become liable for such an array of evils? Our profession brings us constantly in contact with diseased children, who are rendered so—poor little creatures—through the sins of their parents. It would make your hearts ache to witness the sufferings so imposed, and through no fault of the innocent little ones. We fear, that among the very many short-comings the Almighty will call upon us to render an account for, the enfeebled constitutions implanted in our children, through alcoholic and other indescretions, will not be found among the least.

Statistics of our insane asylums show that, but one woman becomes insane to 7.5 men! The explanation of this discrepency exists in the fact that men are more given to severe mental strain and the consequent use of stimulating drinks. It is also shown that paralysis rarely appears under 20 years of age—save in case the child has inebriates for parents—but that it is most common between the ages of 30 and 45, the precise period of immoderate, habitual drinking.

Now, does all this pay? Can we afford, for the sake of an hour's stimulation and hilarity of spirits, to enfeeble our healths, endanger our lives, as well as maiming those of our innocent children?

